# (+)*-epi*-Epoformin, a Phytotoxic Fungal Cyclohexenepoxide: Structure Activity Relationships

**DOI:** 10.3390/molecules23071529

**Published:** 2018-06-25

**Authors:** Antonio Cala, Marco Masi, Alessio Cimmino, José M. G. Molinillo, Francisco A. Macias, Antonio Evidente

**Affiliations:** 1 Allelopathy Group, Department of Organic Chemistry, School of Science, Institute of Biomolecules (INBIO), University of Cádiz, C/República Saharaui 7, 11510 Puerto Real, Cádiz, Spain; antonio.cala@uca.es (A.C.); chema.gonzalez@uca.es (J.M.G.M.); 2 Department of Chemical Sciences, University of Naples “Federico II”, Complesso Universitario Monte S. Angelo, Via Cintia 4, 80126 Napoli, Italy; marco.masi@unina.it (M.M.); alessio.cimmino@unina.it (A.C.); evidente@unina.it (A.E.)

**Keywords:** *Diplodia quercivora*, oak, *epi*-epoformin, cyclohexeneoxide, etiolated wheat coleoptile bioassay, phytotoxicity, allelopathy, SAR

## Abstract

(+)-*epi*-Epoformin (**1**), is a fungal cyclohexene epoxide isolated together with diplopimarane and sphaeropsidins A and C, a *nor*-*ent*-pimarane and two pimaranes, from the culture filtrates of *Diplodia quercivora*, a fungal pathogen for cork oak in Sardinia, Italy. Compound **1** possesses a plethora of biological activities, including antifungal, zootoxic and phytotoxic activity. The last activity and the peculiar structural feature of **1** suggested to carry out a structure activity relationship study, preparing eight key hemisynthetic derivatives and the phytotoxicity was assayed. The complete spectroscopic characterization and the activity in the etiolated wheat coleoptile bioassay of all the compounds is reported. Most of the compounds inhibited growth and some of them had comparable or higher activity than the natural product and the reference herbicide Logran. As regards the structure-activity relationship, the carbonyl proved to be essential for their activity of **1**, as well as the conjugated double bond, while the epoxide could be altered with no significant loss.

## 1. Introduction

**Figure 1:**
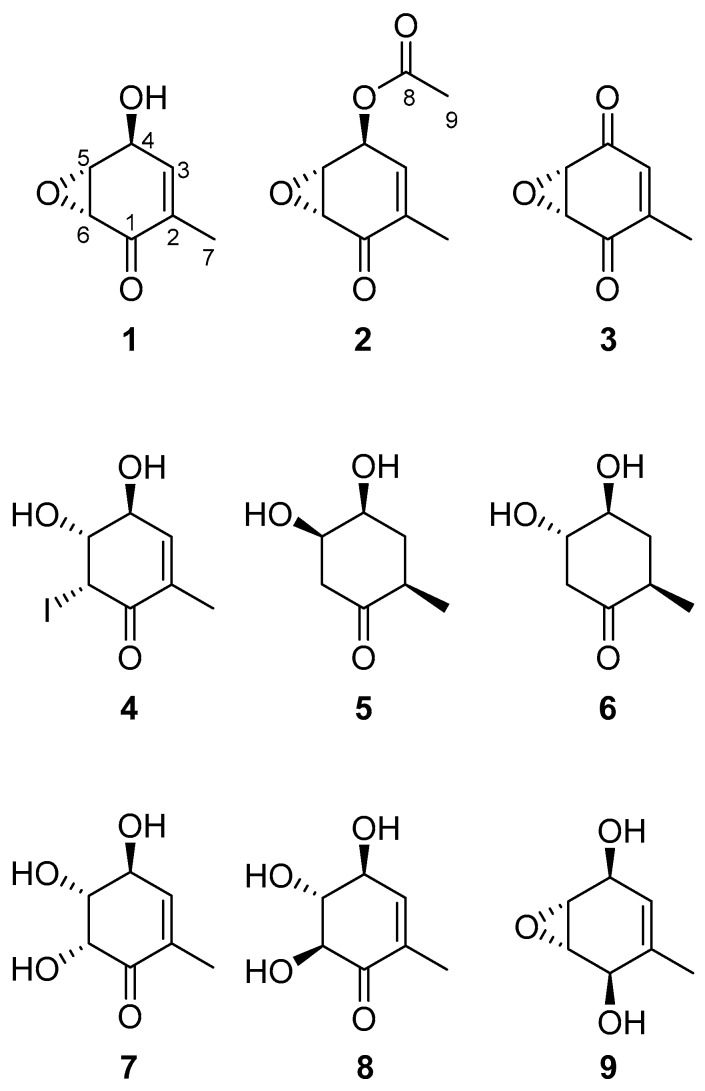
Structure of (+)-*epi*-epoformin (**1**) and derivatives (**2**–**9**).

(+)-*epi*-Epoformin (**1**, Figure 1) is a cyclohexene epoxide, isolated together with some *nor*-*ent*-pimaranes and pimarane diterpenes, namely diplopimarane and sphaeropsidins A and C, from culture filtrates of the fungus *Diplodia quercivora*, isolated from declining *Quercus canariensis* trees in Tunisia as a canker-causing agent [1]. The fungi belonging to *Diplodia* genus are well known as pathogens of forest plants and as producers of several bioactive metabolites belonging to different classes of natural compounds [2].

Compound **1** was also previously reported from an unidentified fungus isolated from a diseased leaf of crepe myrtle (*Lagerstroemia indica* L. Pers.) [3] and showed inhibition of lettuce seed germination. Its total enantioselective and asymmetric synthesis [4,5,6,7] was also developed and the assignment of its absolute configuration was determined by time-dependent density together with that of other close naturally occurring cyclohexene oxides [8].

When isolated from *D. quercivora*, **1** was tested for its phytotoxic activity using different bioassays. In leaf-puncture tests on holm oak (*Quercus ilex* L.), cork oak (*Quercus suber* L.), and tomato (*Lycopersicum esculentum* Mill.) leaves, **1** caused necrotic lesions on holm and cork oak (area lesion sizes of 6.7 and 9.5 mm^2^, respectively), while inducing irregular necrotic lesions (13.5 mm^2^ area) on tomato. In a tomato cutting bioassay, **1** caused, to different extents, stewing on stem at 0.2 and 0.1 mg/mL. It also showed activities against phytopathogenic fungi and oomycetes *Phytophthora cinnamomi* and *Phytophthora plurivora* were the most sensitive target organisms followed by *Athelia rolfsii*, whereas *Diplodia corticola* was the most resistant as its mycelial growth was only slightly inhibited (39.7% inhibition) [1]. Finally, when assayed on brine shrimps (*Artemia salina* L.) **1** caused more than 50% larval mortality at 200 μg/ mL after 36 h to metabolites exposure [1].

More recently, **1** was also used in a preliminary screening to evaluate the potential of plant and fungal metabolites, belonging to different classes of natural compounds, for the biological control of several rusts species belonging to the genera *Puccinia* and *Uromyces*, which are pathogens of very important crops including legumes. Compound **1** showed significant inhibition of all the species tested also exhibiting an effective penetration [9]. The potential fungicide activity of **1** also in plants against *U. pisi* and *P. triticina* was successively confirmed [10].

In this manuscript, **1** was isolated and tested with eight hemisynthetic derivatives (**2**–**9**) in order to obtain clues about the structure-activity (SAR) requirements of these compounds, where we acetylated and oxidized the hydroxy group, open the epoxide, reduce the carbonyl group or saturated the double bond.

## 2. Results and Discussion

The structure-activity relationship study of (+)-*epi*-epoformin (**1**) was carried out preparing eight of its hemisynthetic derivatives. Although **1** was previously described in the literature as a fungal phytotoxic compound [11], there is no information available regarding its activity in etiolated wheat coleoptiles. Thus, the phytotoxic activity of the parent compound (**1**) and its eight hemisynthetic derivatives was assayed using this test.

The culture filtrates of *D. quercivora* were extracted and purified as reported in detail in the Section 3.2. Compound **1** was obtained as a white solid and identified comparing its physical ([α${]}_{D}^{20}$) and spectroscopic (^1^H and ^13^C-NMR and ESI-MS) properties with those previously reported [1,3].

The acetylation of **1** to obtain **2**, in a moderate yield (67%), was successfully carried out using acetic anhydride [12]. Its NMR spectra (Section 3.4) showed that an acetyl group was incorporated to the compound by the new singlet at δ 2.13 (H-9) in the ^1^H-NMR and the corresponding carbons of the acetyl group in the ^13^C-NMR [13], with a carbonyl at δ 193.5 (C-8) and the carbon of the methyl at δ 20.7. A significant downfield shift (from δ 4.67 to δ 5.72) was also observed for H-4 when the ^1^H-NMR spectrum of **1** was compared to that of **2** [14]. In addition, the OH broad band of **1** disappeared in the FTIR spectrum. 2D NMR experiments [15] allowed confirming the structure of **2**. HSQC spectrum was employed to assign each carbon to their corresponding hydrogens and the HMBC showed the correlations to elucidate the position of the acetyl group: the signal at δ 2.13 (H-9) correlated with that at δ 64.5 (C-4), while the signal at δ 5.72 (H-4) correlated with that at δ 169.8 (C-8).

On the other hand, the oxidation of the hydroxyl group with Dess-Martin periodinane [16] was repeated several times changing the reaction times. The highest yield was 44% for **3**, which was obtained after 16 hours, recovering a 51% of the starting material (**1**). A longer reaction time did not significantly increase the yield and less starting material would be recovered. The success on synthesizing **3** was confirmed firstly by the absence of any hydroxyl group and the presence of two narrow bands at 1737 and 1715 cm^−1^ corresponding to the two conjugated carbonyls in the FTIR spectrum [17]. The NMR experiments showed two signals of conjugated carbonyl carbons in the ^13^C-NMR at δ 192.3 (C-1) and 191.2 (C-4) and the signal of the H-4 of **1** disappeared in the ^1^H-NMR. In the HMBC the correlation of the signal at δ 192.3 (C-1) with the methyl signal at δ 2.02 (H-7) was observed allowing to assign that signal to the C-1. Due to the proximity of the signals at δ 54.1 (C-5) and δ 53.7 (C-6) the HSQC experiment was indispensable to assign them to their corresponding signals in the ^1^H-NMR at δ 3.79 (H-5) and δ 3.84 (H-6). These signals were assigned to their positions in the compounds thanks to the correlations in the COSY experiment, where the signal at δ 3.79 (H-5) correlated with the signal at δ 6.43 (H-3).

Following the procedure described by Nicolaou et al. [18] we attempted the opening of the epoxide to obtain a dihydroxylated compound in C-4 and C-5. Unexpectedly, the integrals of the ^1^H-NMR of the product showed that there was only one hydrogen at C-6 at δ 4.77 (H-6), too high for hydrogens in α position to the carbonyl at C-1. After studying all the NMR spectra we determined that the structure was that of **4**, obtained with a high yield (83%), but the compound did not ionize properly to observe the mass corresponding to the product. The presence of iodine in the compound was confirmed by the presence of an I^−^ ion in the MS (ES^−^), with *m*/*z* 126.9045, acting as a leaving group in an intramolecular nucleophilic substitution, which leads to the corresponding neutral epoxide. The stereochemistry of the hydroxyl at C-5 was confirmed by the coupling constants value of H-5 at δ 2.80 with δ 4.77 (H-6) and δ 4.21 (H-4) being *J*_5,6_ = 4.1 Hz (equatorial-equatorial) and *J*_4,5_ = 7.7 Hz (axial-axial).

In order to study the influence of the double bond at C-2 in the bioactivity of **1**, this compound was treated with carbon supported palladium. After several tries, only traces of the expected compound with the epoxide at C-5 and the reduced double bond at C-2 were found. Instead, the major compounds were, unexpectedly, **5** and **6**, with different stereochemistry at C-5 and derived from the reductive opening of the epoxide that occurred at the same time that the reduction of the double bond. By using carbon supported platinum **5** and **6** were again the major products. Interestingly, by using Pd/C the yields were slightly higher for obtaining compounds **5** and **6** (29 and 33%, respectively) than by using Pt/C (19% and 21%, respectively). Only by direct infusion and solving the compounds in MeOH (0.1% formic acid) was possible to ionize these compounds, obtaining the [M + Na]^+^ ions (*m*/*z* 167.3 for C_7_H_12_O_3_Na). The lack of conjugation with a double bond in **5** and **6** moved the carbonyl bands to higher wavenumbers in the FTIR spectrum, from the starting 1674 cm^−1^ in **1** to 1704 and 1711 cm^−1^ in **5** and **6**, respectively. The ^1^H-NMR spectra of both compounds showed that the typical signal of H_2_-3 moved to higher field, being located at δ 1.99 in **5** and δ 2.22 and 1.35 in **6**. Although the stereochemistry of the hydroxyl at H-5 could be assigned by using the values of coupling constants of H-5 with H-4 in both compounds (*J*_4,5_ = 4.3 Hz in **5** and *J*_4,5_ = 8.8 Hz in **6** indicating equatorial-axial and axial-axial couplings, respectively), NOE- and ROE-difference experiments were needed to assign the relative position of the methyl at C-2. By irradiating H-2, H-4 and H-5 in **5** with a ROE-1D experiment, it was confirmed that H-2 (δ 2.78) was close in space to H-4 (δ 4.02) and H-5 (δ 4.18). In addition, H-4 was close in the space to H-6a (δ 2.43). On the other hand, H-4, H-5 and H-3b were irradiated in **6** with a NOE-1D experiment. In this case, it was observed that the signal for H-3b (δ 1.35) was close in space with H-5 (δ 3.65). The latter was close to H-6b (δ 2.75) and H-4 (δ 3.88) was close in space to H-2 (δ 2.46) and H-3a (δ 2.22), thus confirming the opposite stereochemistry of H-5 in **6**. There was one signal for each hydrogen at C-6 in the ^1^H-NMR of both compounds (δ 2.91 and δ 2.43 for **5**, and δ 2.75 and δ 2.46 for compound **6**). In the case of **6**, this was also observed for the hydrogens at C-3 (δ 2.22 and δ 1.35). The COSY and the HSQC experiments were used to assign each pair of signals to their corresponding carbons, while the HMBC allowed differentiating the close carbons at δ 74.0 (C-5, correlated with both H-6) and δ 73.8 (C-4 correlated with both H-3) in **6**.

The opening of the epoxide was performed using basic and acidic media (0.1 M NaOH, 0.1 M HCl and 0.05 M H_2_SO_4_) and none of them worked properly. Surprisingly, while using acyl chlorides with DMAP to acetylate the hydroxyl at C-4 using the Yamaguchi esterification [19], the epoxide opened spontaneously, yielding both compounds **7** and **8** with high yields (39% and 45%, respectively) and no amount of the desired esters. At first, we checked the literature to compare the spectra of our trihydroxylated compounds with the already reported gabosines A and N [20], but the signals were different. Therefore, the spectra were analyzed to determine their structures. Firstly, it was observed the opening of the epoxide in the shift of the signals to lower fields, from δ 3.78 (H-5) and δ 3.53 (H-6) to δ 4.05 and 4.57 (**7**) or δ 3.84 and 4.38 (**8**). H-5 and H-6 were assigned by observing the COSY, where H-5 and H-6 were correlated among themselves, and the HMBC where H-5 correlated with δ 69.3 (C-4), and H-6 correlated with δ 190.6 (C-1) and δ 74.6 (C-5) in the spectrum of **7**. The same correlations were found in **8**, H-5 and H-6 were correlated in the COSY, and in the HMBC H-5 correlated with δ 71.4 (C-4), and H-6 correlated with δ 189.7 (C-1) and δ 78.3 (C-5). Regarding the stereochemistry, the analysis of the NOE-difference experiments and the coupling constants [21] indicated that H-5 and H-6 of **7** were close in the space, H-5 had a high coupling constant with H-4 (*J* = 7.1 Hz) and a lower constant with H-6 (*J* = 3.5 Hz), indicating the 4*S*,5*R*,6*R* stereochemistry. Regarding **8**, the coupling constants of H-5 were with H-4 (*J* = 8.2 Hz) and H-6 (*J* = 11.3 Hz), both high, indicating axial-axial coupling in both cases and the 4*S*,5*R*,6*S* stereochemistry.

Finally, **1** was treated with NaBH_4_ and CeCl_3_ in MeOH to reduce the carbonyl without affecting the double bond or the epoxide, obtaining **9** in a moderate 39% yield. The FTIR showed clearly the presence of an OH broad band at 3234 cm^−1^ and the missing narrow band of the carbonyl. Regarding the ^1^H-NMR, all the signals shifted to a higher field. The lack of the carbonyl was clearly observed in the ^13^C-NMR with only one quaternary signal at δ 135.2 (C-2). The close signals of the epoxide hydrogens and carbons at positions 5 and 6 were assigned with the aid of the COSY, HSQC and HMBC experiments. The signal at δ 3.30 (H-5) correlated with δ 3.36 (H-6) in the COSY, with δ 51.9 in the HSQC, and was a dd with *J* = 3.6 (H-5 with H-4) and 1.5 Hz (H-5 with H-6). On the other hand, the signal at δ 3.36 (H-6) correlated with the signals at δ 3.30 (H-5) and δ 4.25 (H-1) in the COSY, with signals at δ 53.0 (C-6) in the HSQC, and with δ 135.2 (C-2) in the HMBC and was a dd with *J* = 3.5 (H-6 with H-1) and 1.5 Hz (H-6 with H-5). The signal for H-1 was assigned at δ 4.25 after the assignment of the signal at δ 4.46 to H-4 by observing the correlation of δ 4.46 (H-4) with δ 5.51 (H-3) in the COSY. The stereochemistry was assigned using the NOESY experiment, where δ 4.46 (H-4) and δ 4.25 (H-1) where correlated, meaning that they were close in the space. These data confirm the structure and stereochemistry shown in Figure 1 for **9**.

### SAR Study

**Figure 2:**
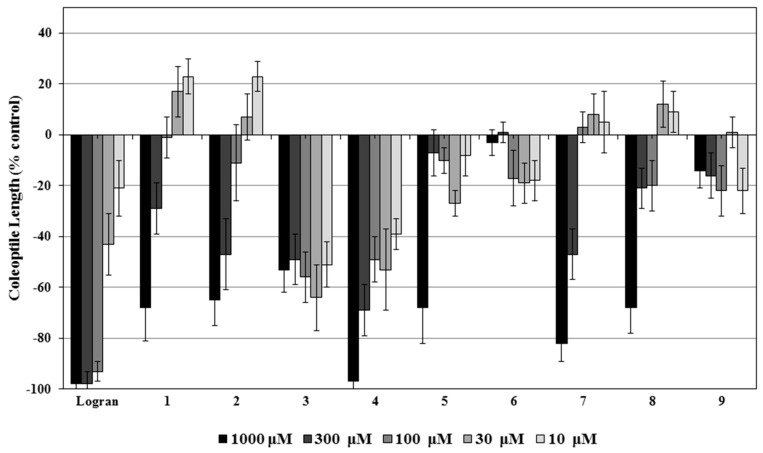
Activity of compounds **1**–**9** in the etiolated wheat coleoptile bioassay. Results are given in % from the control coleoptile length. Positive values indicate a higher growth than the control and negative values a lower growth than the control.

The results from the etiolated wheat coleoptile bioassay of compounds **1**–**9** are shown in Figure 2. In the case of **1** and **2**, a stimulatory activity was observed at 30 and 10 μM. It has been reported that allelopathic compounds can stimulate growth at lower doses and inhibit growth at higher doses [22,23]. According to their IC_50_ values (Table 1), the most active compounds were **3** and then **4**. Compound **3** was one order of magnitude more active than **4** and comparable to the synthetic herbicide Logran^®^, however, the activity profile was inconsistent with the increasing concentration. The IC_50_ for **3** was found at the lowest concentration tested (10 μM) and this value did not change significantly at higher concentrations. The test solutions of **3** were clear and no precipitate was observed; however, this profile is typically linked with a problem of solubility at the highest concentration. It is unknown if this or the nature of the compound caused the strange profile.

Compound **4**, containing iodine in the structure was the most active and three times more active than the next compounds **2** and **7**. The halogen in this structure seems to have a key role in these results, since the other dihydroxylated compound with the same stereochemistry (**6**) and another one with different stereochemistry (**5**) were much less active or not active at all. However, the lack of a double bond removing the α-β-unsaturated system could also play a role. Since the reaction for removing the iodine did not work, we could not explore further this hypothesis.

Alog*P* values varied between −0.88 and 1. Only compounds **2** and **4** followed the Lipinski’s rule of five [24,25], having the appropriate molecular weight and Alo*gP*. As predicted by Lipinski, these two compounds were part of the most active compounds of the collection. However, **7**, violating both requirements, also exhibited a remarkable activity. The stereochemistry seems to play an important role in this compound, since its epimer **8** had almost half the activity.

The least active compounds were **5**, **6** and **9**, showcasing that the double bond and the carbonyl are both indispensable for the observed activity. The epoxide, on the other hand, proved to be superfluous, since the opening of the epoxide did not result in a drop in activity (compounds **4**, **7** and **8**) and the compound preserving the epoxide and lacking the carbonyl had nil activity (**9**).

In summary, the addition of iodine in the molecule (compound **4**) and an extra hydroxyl with the appropriate stereochemistry (compound **7**) had a positive effect in the bioactivity, while the acetylation (compound **2**) of the hydroxyl naturally present had little effect and the reduction of the carbonyl (compound **9**) or the double bond (compounds **5** and **6**) had a negative effect.

A possible explanation of the higher activity of derivatives **3** and **4** was that **3** has a hemiquinone structure and derivative **4** by elimination of HI via E2 could be converted into the corresponding hemiquinone derivative and thus in the corresponding quinone. This could also occur in derivative **7** but the leaving group from C-6 is OH^−^ with respect to the good one I^−^ in **4**. The hemiquinone generated from **7**, being also an enolic compound could be stabilized from the hydrogen bond between OH-6 and O=C-1. Because of the low oxidation level of derivatives **2**, **5**, **6**, **8** and **9**, as well as **1**, they would be more difficult to convert in the corresponding hemiquinone.

This hypothesis on the mode of action of *epi*-epoformin is in full agreement with the SAR study carried out using some derivatives of sphaeropsidones, two epimeric phytotoxic cyclohexene epoxides isolated from *Diplodia cupressi* (a cypress pathogen in the Mediterranean basin) [26] and the testing their phytotoxic and antifungal activities on non-host plants and on five fungal pathogenic species belonging to the genus Phytophthora [27]. Successively, when sphaeropsidones and the same and new other derivatives were tested by haustorium-inducing activity in *Orobanche cumana*, *O. crenata* and *Striga hermonthica*, the results obtained suggested that the activity is due to the possibility to convert the natural sphaeropsidones and natural and hemisynthetic derivatives into the corresponding 3-methoxyquinone [28].

This hypothesis on the mode of action of sphaeropsidone, that as above reported could work also for *epi*-epoformin, is in full agreement with the results obtained using natural and synthetic quinones as sorghum xenognosin and dimethoxybenzoquinones, in studies carried out on haustoria and the chemistry in host recognition parasitic angiosperms. Quinone/hydroquinone structures serve as cofactors in many metabolic pathways, playing critical chemical roles in oxidation/reduction processes [29,30].

## 3. Materials and Methods

### 3.1. General Experimental Procedures

The purities of the compounds were determined by ^1^H-NMR spectroscopy and every compound was purified in the HPLC prior bioassays. ^1^H-NMR and ^13^C-NMR spectra were recorded on Agilent™ (Santa Clara, CA, USA) 400 and 500 MHz spectrometers using CDCl_3_ (MagniSolv™, Merck, Darmstadt, Germany) as solvent. The residual peaks of the solvent at 7.26 ppm (^1^H-NMR) and 77.0 ppm (^13^C-NMR) were used as the internal reference. Carbon multiplicities were determined by DEPT spectra [16]. DEPT, COSY, HSQC, HMBC, and NOESY experiments were performed using Varian vnmrj microprograms. Exact masses were measured on a UPLC-QTOF ESI (Waters Synapt G2, Manchester, UK) high-resolution mass spectrometer (HRTOFESIMS). Mass spectra were recorded in the negative- or positive-ion mode in the range *m*/*z* 100–2000, with a mass resolution of 20,000 and an acceleration voltage of 0.7 kV. FTIR spectra were obtained on Perkin-Elmer (Waltham, MA, USA) Spectrum TWO IR spectrophotometer. Major absorptions in the infrared are given as wavenumbers $\tilde{\upsilon }$ in cm^−1^.Optical rotations were measured in CHCl_3_ on a JASCO (Easton, PA, USA) P-2000 polarimeter. HPLC in the isocratic mode was performed on a Merck Hitachi D-7000 equipped with a LiChroCART 250-10 Si 60 (10 μm) column. Column chromatography (CC) was performed using silica gel (Merck, Geduran^®^ Si 60, 0.063–0.200 mm) and C_18_-reversed phase silica gel (Sigma-Aldrich, C_18_ phase 90 A pore size, St. Louis, MO, USA). Analytical and preparative TLC were performed on silica gel (Kieselgel 60, F_254_, 0.25 and 0.5 mm respectively) and on reversed phase (Merck, Kieselgel 60 RP-18, F_254_, 0.20 mm) plates. The spots were visualized by exposure to UV radiation (253 nm), or iodine vapour, or by spraying first with 10% H_2_SO_4_ in MeOH and then with 5% phosphomolybdic acid in EtOH, followed by heating at 110 °C for 10 min. Either Sigma-Aldrich Co., Merck or Alfa Aesar (Ward Hill, MA, USA) supplied the reagents and the solvents. Seeds for the etiolated wheat coleoptile bioassay were kindly supplied by Fitó (Barcelona, Spain).

### 3.2. Fungal Strain

The fungal strain of *D. quercivora* used in this study was originally isolated from a symptomatic branch of *Q. canariensis* Willd. collected in a natural area in Tunisia as previously described [1].

### 3.3. Extraction and Purification of (+)-epi-Epoformin (***1***) from D. quercivora

The fungus was and grown in vitro as previously described [1]. The culture filtrates (6.7 L) were acidified to pH 4 with 2 M HCl and extracted exhaustively with EtOAc. The organic extracts were combined, dried with Na_2_SO_4_, and evaporated under reduced pressure to give a brown oil residue (1.14 g). This latter was fractionated through chromatography column on silica gel, eluted with CHCl_3_:*i*-PrOH (95:5). Eight homogeneous fraction groups were collected. The residue of the fourth fraction (474.6 mg) was purified by CC on reverse phase eluted with Me_2_CO:H_2_O (7:3), yielding **1** (276.1 mg) as a white solid. The spectroscopic data of **1** are as follows: HRMS, *m*/*z* (M^+^) calcd for C_7_H_9_O_3_ 141.0552, found 141.0601 [M + H]^+^; IR $\tilde{\upsilon }$_max_ 3357 (O–H), 1674 (C=O) cm^–1^, [α${]}_{D}^{20}$ = +139.3° (c = 0.08). ^1^H-NMR (400 MHz, CDCl_3_, δ, ppm): 6.46 (brs, 1H, H-3), 4.67 (brs, 1H, H-4), 3.78 (m, 1H, H-5), 3.53 (m, 1H, H-6), 1.86 (s, 3H, H-7).

### 3.4. Synthesis of (+)-epi-Epoformin (***1***) Derivatives ***2***–***9***

**Scheme 1:**
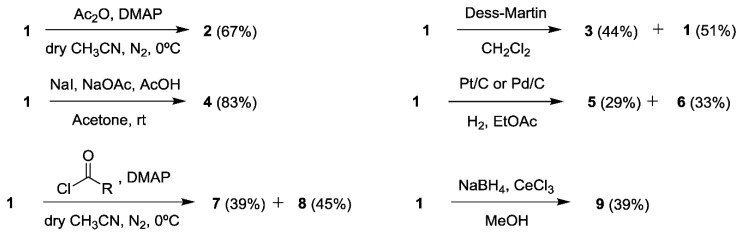
Synthesis of compounds **2**–**9**.

***(****1R,2S,6R)-4-Methyl-5-oxo-7-oxabicyclo[4.1.0]hept-3-en-2-yl acetate* (**2**). Compound **1** (10.8 mg, 0.077 mmol) was dissolved in dry CH_3_CN (2 mL) in a 10 mL pear-shaped flask, in N_2_ atmosphere at 0 °C. Then 4-dimethylaminopyridine (10.5 mg, 0.085 mmol) and acetic anhydride (29 μL, 0.308 mmol) were added and stirred slowly reaching room temperature (Scheme 1). After 10 minutes, the reaction was quenched with 2 mL brine and extracted ×3 with EtOAc. The organic layers were combined, dried with anhydrous Na_2_SO_4_ and concentrated under reduced pressure in a rotatory evaporator obtaining 14.7 mg of crude extract. After purification in column chromatography using *n*-hexane:acetone, 9:1, 9.4 mg of **2** (67% yield) were obtained as a light-yellow oil. Spectroscopic data are as follows: HRMS, *m*/*z* (M^+^) calcd for C_9_H_11_O_4_ 183.0657, found 183.0504 [M + H]^+^; IR $\tilde{\upsilon }$_max_ 2926 (C–H), 1741 (C=O), 1687 (C=O) cm^–1^, [α${]}_{D}^{20}$ = +102.8° (c = 0.13). ^1^H-NMR (500 MHz, CDCl_3_, δ, ppm): 6.37 (m, 1H, H-3), 5.72 (dd, *J* = 5, 1.2 Hz, 1H, H-4), 3.72 (m, 1H, H-5), 3.53 (dd, *J* = 3.6, 1.1 Hz, 1H, H-6), 2.13 (s, 3H, H-9), 1.86 (dd, *J* = 1.4, 1.2 Hz, 3H, H-7). ^13^C-NMR (125 MHz, CDCl_3_, δ, ppm): 193.5 (s, C-1), 169.8 (s, C-8), 136.6 (s, C-2), 134.3 (d, C-3), 64.5 (d, C-4), 55.0 (d, C-5), 52.9 (d, C-6), 20.7 (q, C-9), 16.0 (q, C-7).

*(1R,6S)-3-Methyl-7-oxabicyclo[4.1.0]hept-3-ene-2,5-dione* (**3**). Compound **1** (9.8 mg, 0.070 mmol) was dissolved in CH_2_Cl_2_ (1 mL) in a 10 mL pear-shaped flask. Then Dess-Martin periodinane (45.9 mg, 0.105 mmol) was added in small portions while stirring at rt. After 16 hours, the reaction was quenched with 1 mL of aqueous saturated Na_2_S_2_O_3_ and extracted ×3 with CH_2_Cl_2_. The organic layers were combined and washed with 1 mL aqueous saturated NaHCO_3_, dried with anhydrous Na_2_SO_4_ and concentrated under reduced pressure in a rotatory evaporator. The crude was purified in column chromatography in a gradient of *n*-hexane:EtOAc from 1:0 to 2:3, and later in the HPLC using hexane:acetone 9:1 with a retention time of 12 min. Compound **3** was obtained as a colorless oil in a 44% yield (4.3 mg) and 5 mg of **1** (51%) were recovered. Spectroscopic data are as follows: HRMS, *m*/*z* (M^+^) calcd for C_7_H_7_O_3_ 139.0395 [M + H]^+^, found 139.0401; IR $\tilde{\upsilon }$_max_ 2925 (C–H), 1737 (C=O), 1715 (C=O) cm^–1^, [α${]}_{D}^{20}$ = +5.8° (c = 0.02). ^1^H-NMR (500 MHz, CDCl_3_, δ, ppm): 6.43 (brs, 1H, H-3), 3.84 (dd, *J* = 3.7, 0.7 Hz, 1H, H-6), 3.79 (dd, *J* = 2.5, 0.7 Hz, 1H, H-5), 2.02 (d, *J*= 1.4 Hz, 3H, H-7). ^13^C-NMR (125 MHz, CDCl_3_, δ, ppm): 192.3 (s, C-1), 191.2 (s, C-4), 146.8 (s, C-2), 133.3 (d, C-3), 54.1 (d, C-5), 53.7 (d, C-6), 16.4 (q, C-7).

*(4S,5R,6R)-4,5-Dihydroxy-6-iodo-2-methylcyclohex-2-en-1-one* (**4**). Compound **1** (9.8 mg, 0.070 mmol) was dissolved in acetone (1 mL) in a 10 mL pear-shaped flask. Then NaI (37 mg, 0.252 mmol), NaOAc (2.3 mg, 0.252 mmol) and AcOH (14 μL, 0.252 mmol) were added and stirred at rt. After 7 hours, the reaction was quenched with 1 mL of aqueous saturated Na_2_S_2_O_3_ and evaporated the acetone under reduced pressure. The resultant aqueous solution was extracted ×3 with EtOAc. The organic layers were combined and washed with 1 mL H_2_O, 1 mL aqueous saturated NaHCO_3_ and 1 mL brine, dried with anhydrous Na_2_SO_4_ and concentrated under reduced pressure in a rotatory evaporator. The crude was purified in column chromatography in a gradient of *n*-hexane:EtOAc from 1:0 to 2:3, and furnished 13.8 mg of a fraction with a mixture of two compounds. This fraction was later purified in the HPLC with *n*-hexane:acetone 7:3 obtaining two peaks at 15 (**4**) and 17 min. A total of 8.2 mg of **4** (83% yield) as a colorless oil were isolated. Spectroscopic data are as follows: HRMS, *m*/*z* (M^−^) calcd for I 126.9050, found 126.9045 [M]^−^; IR $\tilde{\upsilon }$_max_ 3264 (O–H), 2925 (C–H), 1683 (C=O) cm^–1^, [α${]}_{D}^{20}$ = +67.3° (c = 0.05). ^1^H-NMR (400 MHz, CDCl_3_, δ, ppm): 6.59 (m, 1H, H-3), 4.77 (d, *J* = 4.1 Hz, 1H, H-6), 4.21 (dt, *J* = 7.7, 2.1 Hz, 1H, H-4), 2.80 (dd, *J* = 7.7, 4.1 Hz, 1H, H-5), 1.87 (dd, *J* = 2.1, 1.5 Hz, 3H, H-7). ^13^C-NMR (100 MHz, CDCl_3_, δ, ppm): 192.3 (s, C-1), 143.9 (d, C-3), 133.8 (s, C-2), 73.6 (d, C-4), 73.5 (d, C-5), 35.2 (d, C-6), 15.9 (q, C-7).

*(2R,4S,5R)-4,5-Dihydroxy-2-methylcyclohexan-1-one (**5**) and (2R,4S,5S)-4,5-dihydroxy-2-methylcyclohexan-1-one (***6***)*. Compound **1** (9.8 mg, 0.070 mmol) was disolved in EtOAc (2 mL) in a 10 mL pear-shaped flask and N_2_ atmosphere. Then carbon supported Pt (23 mg, 0.004 mmol) was added followed by H_2_ atmosphere and a H_2_ balloon was connected while stirring at rt. After 10 minutes, no more starting material was observed in TLC and the crude of reaction was filtered through celite. Then, it was purified by column chromatography with a gradient of *n*-hexane:acetone from 1:0 to 2:3, furnishing a major fraction of 4.4 mg containing the mixture of reduced products. This fraction was purified in the HPLC with *n*-hexane:acetone 7:3 observing two peaks at 25 (**5**) and 32 min. (**6**), with a total mass of 1.9 mg (19% yield) and 2.2 mg (21%), respectively, both being colorless oils. Alternatively, using Pd/C(3 mg, 0.003 mmol) instead of Pt/C, and 9.2 mg of **1** (0.066 mmol), 2.7 mg of compound **6** (29% yield) and 3.2 mg of **6** (33% yield) were obtained. Spectroscopic data of **5** are as follows: MS, *m*/*z* (M^+^) calcd for C_7_H_12_O_3_Na 167.0684, found at low resolution 167.3 [M + Na]^+^; IR $\tilde{\upsilon }$_max_ 3385 (O–H), 2926 (C–H), 1704 (C=O) cm^–1^, [α${]}_{D}^{20}$ = +13.1° (c = 0.03). ^1^H-NMR (400 MHz, CDCl_3_, δ, ppm): 4.18 (ddd, *J* = 4.9, 4.3, 3.8 Hz, 1H, H-5), 4.02 (dd, *J* = 8.4, 4.3 Hz, 1H, H-4), 2.91 (dd, *J* = 14.4, 3.8 Hz, 1H, H-6b), 2.78 (m, 1H, H-2), 2.43 (dd, *J* = 14.4, 4.9 Hz, 1H, H-6a), 1.99 (dd, *J* = 8.4, 4.0 Hz, 2H, H-3), 1.08 (d, *J* = 6.8 Hz, 3H, H-7). ^13^C-NMR (100 MHz, CDCl_3_, δ, ppm): 211.5 (s, C-1), 74.0 (d, C-5), 69.0 (d, C-4), 44.6 (t, C-6), 40.1 (d, C-2), 36.9 (t, C-3), 14.8 (q, C-7). Spectroscopic data of **6** are as follows: MS, *m*/*z* (M^+^) calcd for C_7_H_12_O_3_Na 167.0684, found at low resolution 167.3 [M + Na]^+^; IR $\tilde{\upsilon }$_max_ 3392 (O–H), 2930 (C–H), 1711 (C=O) cm^–1^, [α${]}_{D}^{20}$ = +11.5° (c = 0.03). ^1^H-NMR (400 MHz, CDCl_3_, δ, ppm): 3.88 (ddd, *J* = 13.3, 8.8, 4.5 Hz, 1H, H-4), 3.65 (ddd, *J* = 12.2, 8.8, 5.3 Hz, 1H, H-5), 2.75 (ddd, *J* = 13.7, 5.3 Hz, 1H, H-6b), 2.46 (m, 1H, H-2), 2.46 (m, 1H, H-6a), 2.22 (ddd, *J* = 13.2, 5.7, 4.5 Hz, 1H, H-3a), 1.35 (td, *J* = 13.2, 11.4, 1H, H-3b), 1.06 (d, *J* = 6.6 Hz, 3H, H-7). ^13^C-NMR (100 MHz, CDCl_3_, δ, ppm): 207.9 (s, C-1), 74.0 (d, C-5), 73.8 (d, C-4), 47.0 (t, C-6), 42.4 (d, C-2), 37.1 (t, C-3), 13.8 (q, C-7).

*(4S,5R,6R)-4,5,6-Trihydroxy-2-methylcyclohex-2-en-1-one* (**7**) *and (4S,5R,6S)-4,5,6-trihydroxy-2-methyl-cyclohex-2-en-1-one* (**8**). Compound **1** (7.1 mg, 0.051 mmol) was dissolved in dry CH_3_CN (2 mL) in a 10 mL pear-shaped flask. Then 4-dimethylaminopyridine (6.9 mg, 0.056 mmol) and hexanoyl chloride (29 μL, 0.204 mmol) were added while stirring at 0 °C and gradually reached rt. After 5 hours and 30 min. no more starting material was observed in TLC and the reaction was quenched with 2 mL NaHCO_3_ and extracted ×3 with EtOAc. The organic layers were combined, dried over anhydrous Na_2_SO_4_ and the solvent evaporated in a rotatory evaporator. The crude was purified by column chromatography with a gradient of *n*-hexane:acetone from 1:0 to 2:3, furnishing two fractions. These fractions were purified in the HPLC with *n*-hexane:acetone 7:3 observing two peaks at 16 (**7**) and 17 min. (**8**), with a total mass of 3.2 mg (39% yield) and 3.6 mg (45%), respectively, both as colorless oils. The same compounds and similar yields were obtained when using butanoyl chloride (22 μL, 0.204 mmol) and the same procedure. Spectroscopic data of **7** are as follows: HRMS, *m*/*z* (M^+^) calcd for C_7_H_10_O_4_ 157.0506, found 157.0505 [M − H]^−^; IR $\tilde{\upsilon }$_max_ 3372 (O–H), 2926 (C–H), 1682 (C=O) cm^–1^, [α${]}_{D}^{20}$ = +65.7° (c = 0.03). ^1^H-NMR (400 MHz, CDCl_3_, δ, ppm): 6.68 (dq, *J* = 2.3, 1.4 Hz, 1H, H-3), 4.59 (ddd, *J* = 7.1, 2.3, 2.1 Hz, 1H, H-4), 4.57 (d, *J* = 3.5 Hz, 1H, H-6), 4.05 (d, *J* = 7.1, 3.5 Hz, 1H, H-5), 1.88 (dd, *J* = 2.1, 1.4 Hz, 3H, H-7). ^13^C-NMR (100 MHz, CDCl_3_, δ, ppm): 190.6 (s, C-1), 144.1 (d, C-3), 134.3 (s, C-2), 74.6 (d, C-5), 69.3 (d, C-4), 61.4 (d, C-6), 15.7 (q, C-7). Spectroscopic data of **8** are as follows: HRMS, *m*/*z* (M^+^) calcd for C_7_H_10_O_4_ 157.0506, found 157.0540 [M − H]^−^; IR $\tilde{\upsilon }$_max_ 3370 (O–H), 2925 (C–H), 1691 (C=O) cm^–1^, [α${]}_{D}^{20}$ = +28.2° (c = 0.03). ^1^H-NMR (400 MHz, CDCl_3_, δ, ppm): 6.70 (dq, *J* = 2.1, 1.5 Hz, 1H, H-3), 4.51 (ddd, *J* = 8.2, 2.1, 2.1 Hz, 1H, H-4), 4.38 (d, *J* = 11.3 Hz, 1H, H-6), 3.84 (d, *J* = 11.3, 8.2 Hz, 1H, H-5), 1.89 (dd, *J* = 2.1, 1.5 Hz, 3H, H-7). ^13^C-NMR (100 MHz, CDCl_3_, δ, ppm): 189.7 (s, C-1), 144.2 (d, C-3), 135.1 (s, C-2), 78.3 (d, C-5), 71.4 (d, C-4), 66.9 (d, C-6), 15.8 (q, C-7).

*(1S,2R,5S,6R)-3-Methyl-7-oxabicyclo[4.1.0]hept-3-ene-2,5-diol* (**9**). Compound **1** (9.8 mg, 0.070 mmol) was dissolved in MeOH (2 mL) containing 0.4 M CeCl_3_ (0.8 mmol) in a 10 mL pear-shaped flask. Then NaBH_4_ (3.8 mg, 0.102 mmol) were added in small portions and stirred at rt. The reaction was quenched after 5 min. with 2 mL saturated aqueous NH_4_Cl, evaporated the MeOH in a rotatory evaporator and extracted ×3 with EtOAc. The organic layers were combined, dried over anhydrous Na_2_SO_4_ and the solvent evaporated. Product was purified in the HPLC with *n*-hexane:acetone 7:3 observing a peak at 34 min., corresponding to **9**, with a total mass of 3.8 mg (39% yield) as an amorphous colorless solid. Spectroscopic data of **9** are as follows: HRMS, *m*/*z* (M^+^) calcd for C_7_H_11_O_3_ 143.0708, found 143.0681 [M + H]^+^; IR $\tilde{\upsilon }$_max_ 3234 (O–H), 2916 (C–H) cm^–1^, [α${]}_{D}^{20}$ = +0.8° (c = 0.03). ^1^H-NMR (400 MHz, CDCl_3_, δ, ppm): 5.51 (dd, *J* = 5.1, 1.6 Hz, 1H, H-3), 4.46 (ddd, *J* = 5.1, 3.6, 0.9Hz, 1H, H-4), 4.25 (d, *J* = 3.5, 1.5 Hz, 1H, H-1), 3.36 (dd, *J* = 3.5, 1.5 Hz, 1H, H-6), 3.30 (dd, *J* = 3.6, 1.5 Hz, 1H, H-5), 1.83 (dd, *J* = 1.6, 0.9 Hz, 3H, H-7). ^13^C-NMR (100 MHz, CDCl_3_, δ, ppm): 135.2 (s, C-2), 121.6 (d, C-3), 66.3 (d, C-1), 63.1 (d, C-4), 53.0 (d, C-6), 51.9 (d, C-5), 21.2 (q, C-7).

^1^H- and ^13^C-NMR Spectra of the compounds are available in the Supplementary Materials.

### 3.5. Etiolated Wheat Coleoptile Bioassay

The compounds **1**–**9** were tested for their bioactivity in an etiolated wheat coleoptile bioassay. The conditions for this bioassay were reported previously [31,32] and replicated in this study, using the same herbicide (Logran^®^) as positive control and the buffer solution as negative control. Wheat (*Triticum aestivum*) was the ‘catervo’ variety. All the samples were solved in a 0.5% of dimethyl sulfoxide and gave clear solutions at all the tested concentrations (10^−3^–10^−5^ M). The results are shown in Figure 2.

### 3.6. Calculation of IC_50_ and logP

The bioactivity data were fitted to a sigmoidal dose-response model using the GraphPad Prism v.5.00 software package (GraphPad Software, San Diego, CA, USA) [33] to obtain the IC_50_ values shown in Table 1. The Alog*P* was calculated using the ALOGPS v.2.1 software (Helmholtz Zentrum München, Munich, Germany), based on computed atom contributions [34,35,36].

## 4. Conclusions

(+)-*epi*-Epoformin (**1**) was successfully isolated from extracts of *D. quercivora* following the procedures in the literature. Then, eight derivatives of **1** were synthesized and tested altogether for their bioactivity in the etiolated wheat coleoptile bioassay. The derivatives were compared with the starting material to look for structure-activity relationships. The α-β-unsaturated system in **1** proved to be essential to obtain active derivatives and the inclusion of iodine and an extra hydroxyl in the molecules increased the activity, while the acetylation of the hydroxyl did not have a significant effect. The results described here illustrate that natural products, especially those provided by fungi, are valuable resources which need to be investigated further as natural herbicides against weeds.

## Figures and Tables

**Table 1:** IC_50_ and Alog*P* values for compounds **1**–**9**. IC_50_ was calculated only for those that reached the 50% of inhibition in the bioassay. ^a^ Tested concentration at which the inhibition was −50%. ^b^ Tested compound which not reached 50% inhibition.

Compound	Alog*P*	IC_50_ (μM)	R^2^
1	−0.12	457	0.9737
2	0.26	326	0.9630
3	0.13	10.0 ^a^	-
4	1.00	117	0.9913
5	−0.43	762	1.000
6	−0.43	- ^b^	-
7	−0.88	307	0.9887
8	−0.88	580	0.9916
9	−0.36	- ^b^	-
Logran	-	33.1	0.9898

## Supplementary Materials

Supplementary data are available online.
