# Supplementary material for: (+)-epi-Epoformin, a Phytotoxic Fungal Cyclohexenepoxide: Structure Activity Relationships
Source: Molecules. 2018 Jun 25;23(7):1529. doi: 10.3390/molecules23071529 (PMC6100255; doi:10.3390/molecules23071529)
Supplement: Supplementary file 1 [file molecules-23-01529-s001.pdf]

SUPPORTING INFORMATION

(+)-*epi*-Epoformin, a phytotoxic fungal cyclohexenepoxide: Structure activity relationships

Antonio Cala[1, †], Marco Masi[2, †], Alessio Cimmino[2], José M.G. Molinillo[1], Francisco A. Macias[1] and Antonio Evidente[2]

1 Allelopathy Group, Department of Organic Chemistry, School of Science, Institute of Biomolecules (INBIO), University of Cádiz, C/ República Saharaui 7, 11510-Puerto Real, Cádiz, Spain; antonio.cala@uca.es (A.C.); chema.gonzalez@uca.es (J.M.G.M.), famacias@uca.es (F.A.M.).
2 Department of Chemical Sciences, University of Naples "Federico II", Complesso Universitario Monte S. Angelo, Via Cintia 4, 80126 Napoli, Italy; marco.masi@unina.it (M.M.); alessio.cimmino@unina.it (A.C.), evidente@unina.it (A.E.).

† These authors contributed equally to this work.

*Correspondence:

Table of Contents

**S3.**	**^{1}H NMR Spectrum of 1**
**S4.**	**^{1}H and ^{13}C NMR Spectra of 2**
**S5.**	**^{1}H and ^{13}C NMR Spectra of 3**
**S6.**	**^{1}H and ^{13}C NMR Spectra of 4**
**S7.**	**^{1}H and ^{13}C NMR Spectra of 5**
**S8.**	**^{1}H and ^{13}C NMR Spectra of 6**
**S9.**	**^{1}H and ^{13}C NMR Spectra of 7**
**S10.**	**^{1}H and ^{13}C NMR Spectra of 8**
**S11.**	**^{1}H and ^{13}C NMR Spectra of 9**

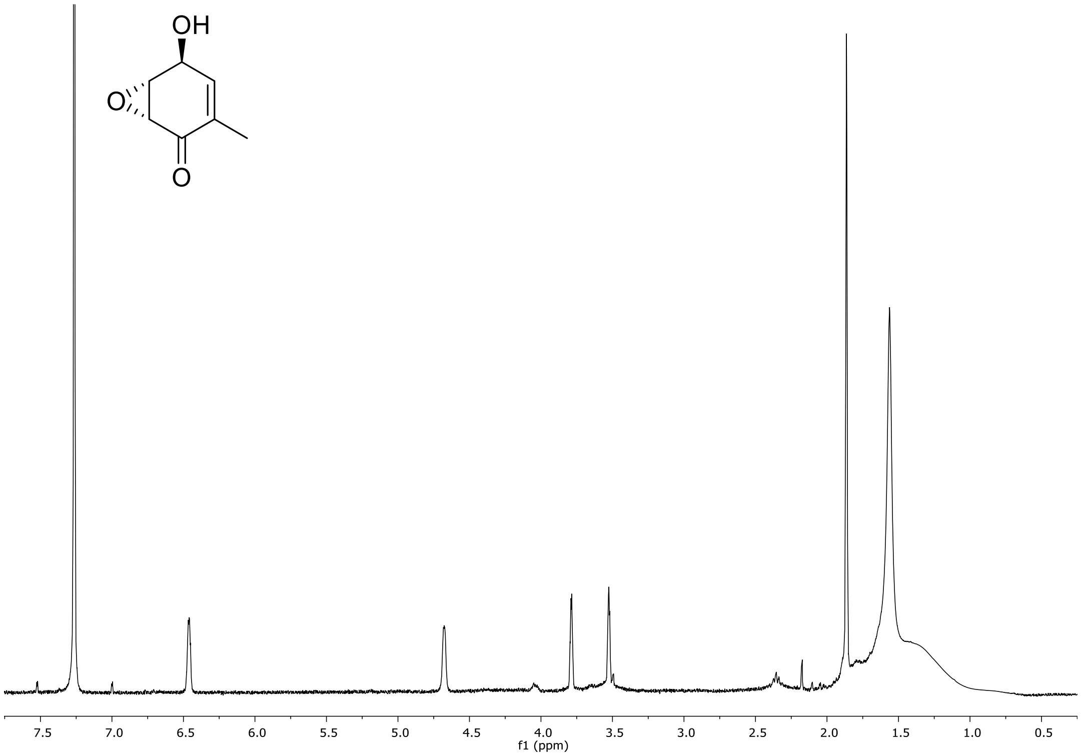

^{1}H-NMR Spectrum of **1** (400MHz, $CDCl_3$)

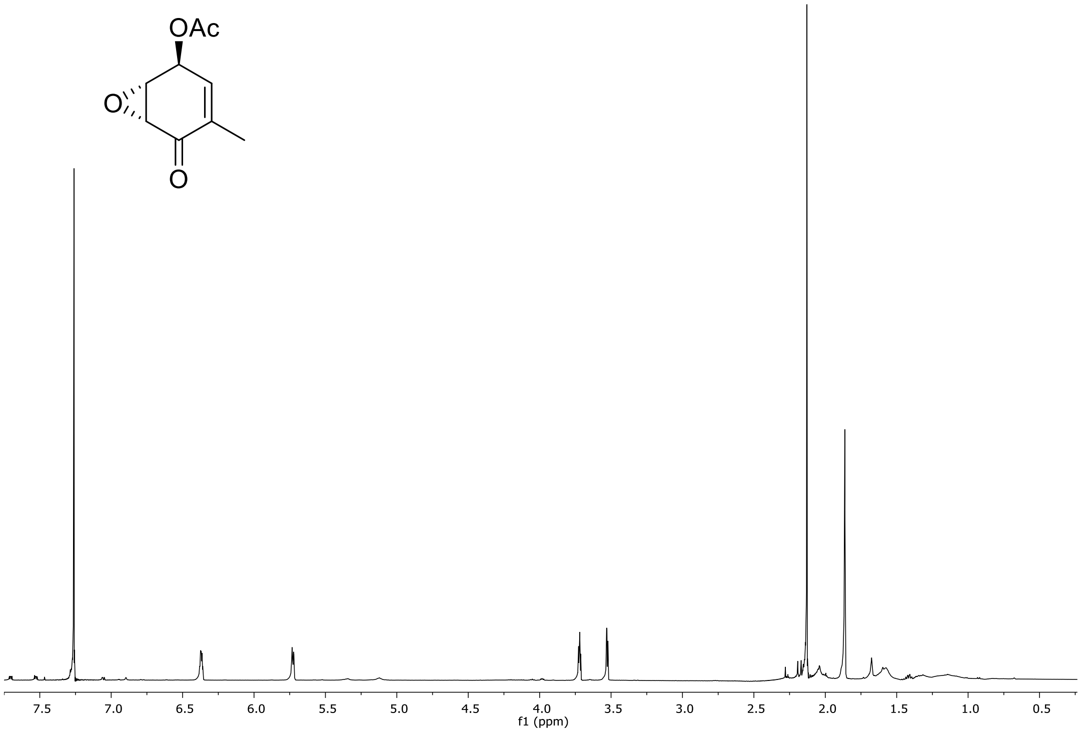

^{1}H-NMR Spectrum of **2** (500MHz, $CDCl_3$)

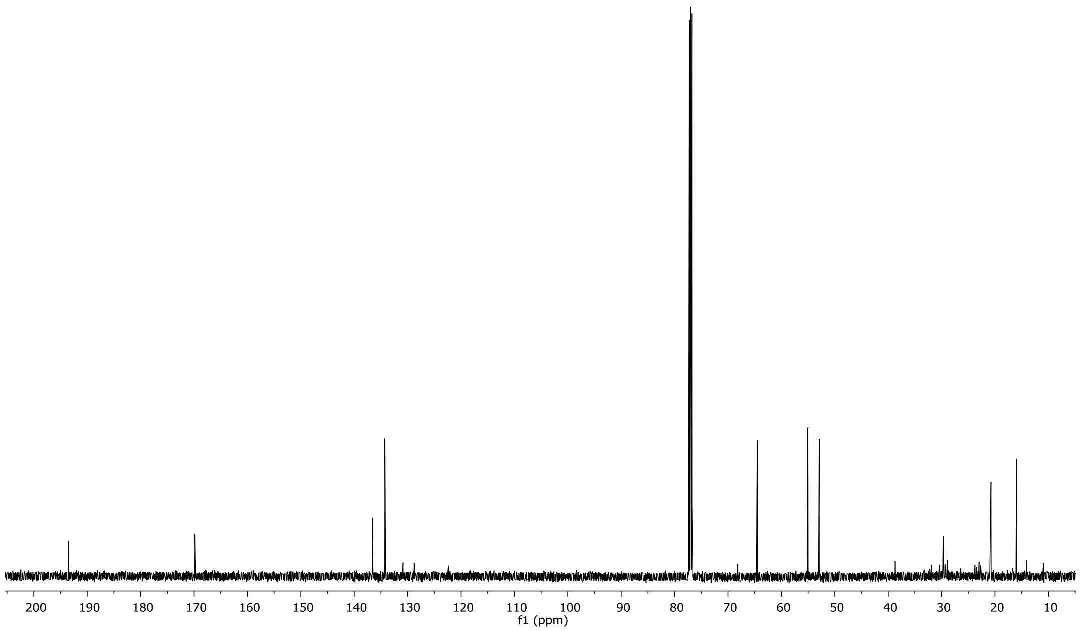

^{13}C-NMR Spectrum of **2** (125MHz, $CDCl_3$)

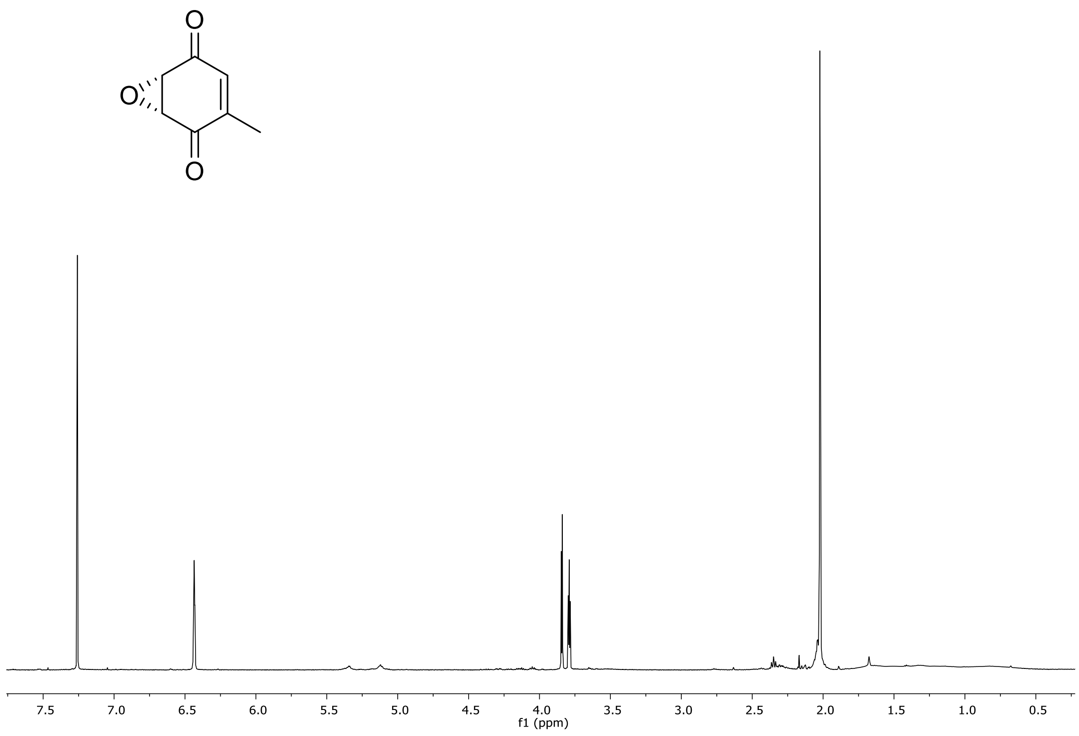

^{1}H-NMR Spectrum of **3** (500MHz, $CDCl_3$)

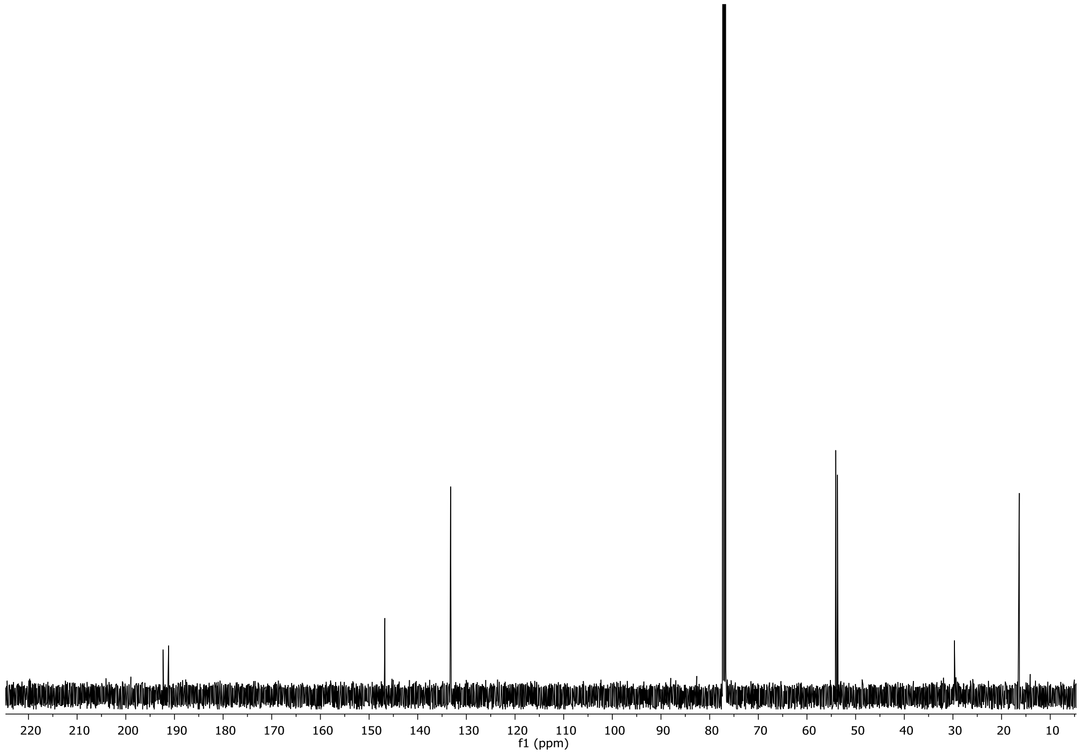

^{13}C-NMR Spectrum of **3** (125MHz, $CDCl_3$)

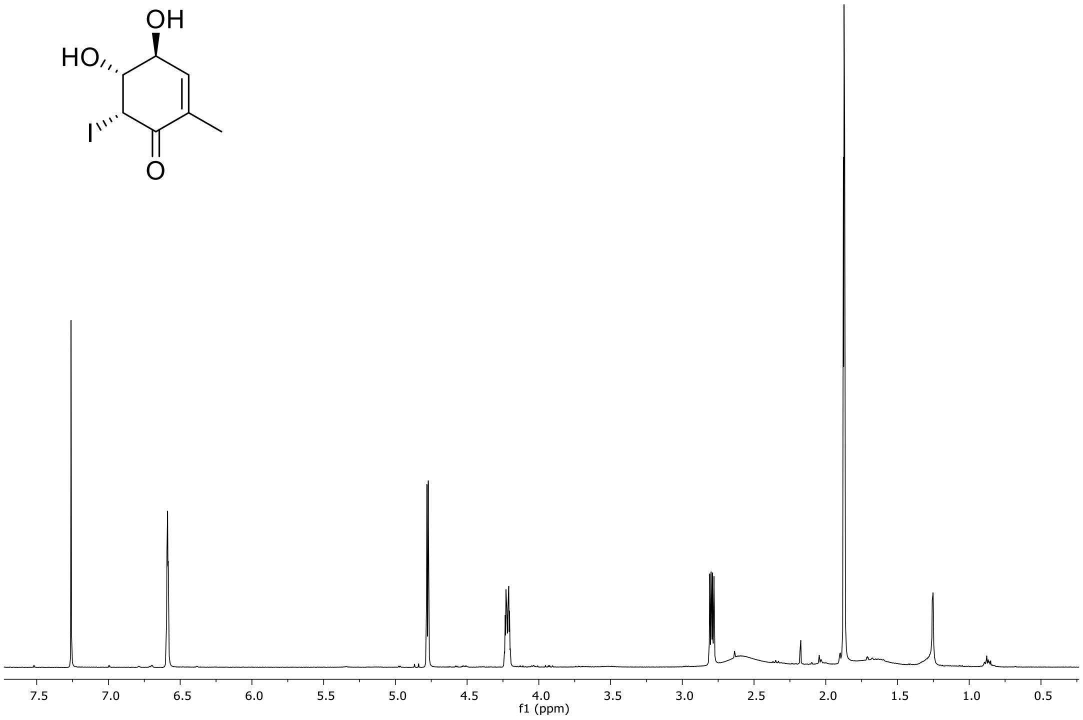

^{1}H-NMR Spectrum of **4** (400MHz, $CDCl_3$)

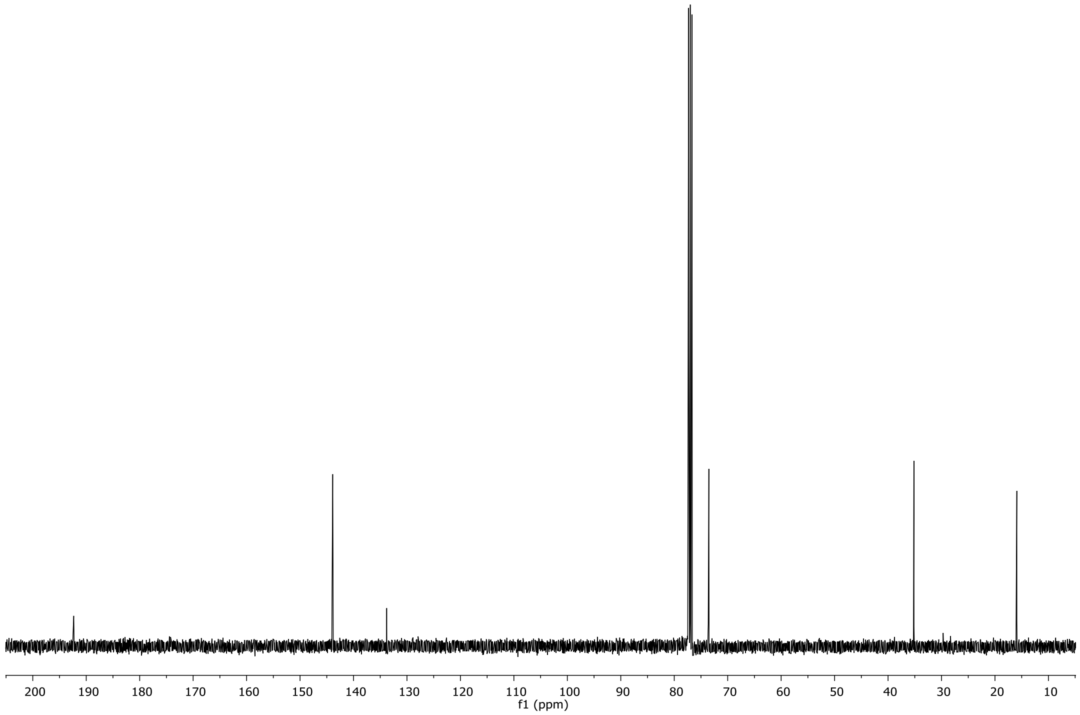

^{13}C-NMR Spectrum of **4** (100MHz, $CDCl_3$)

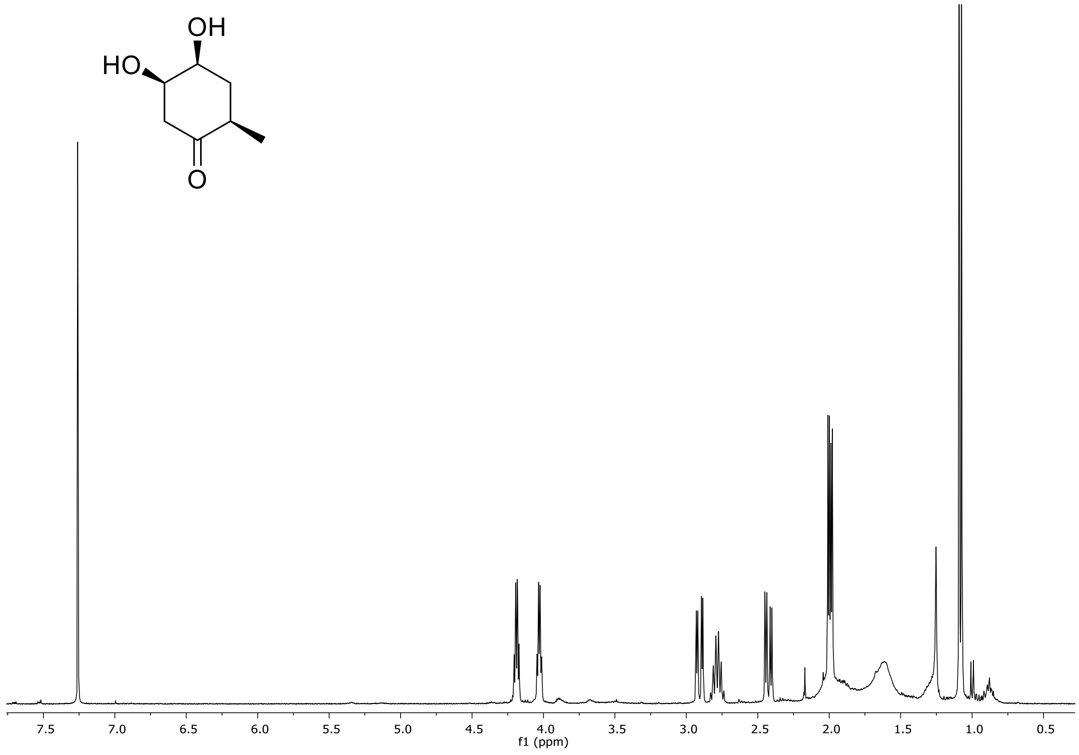

^{1}H-NMR Spectrum of **5** (500MHz, $CDCl_3$)

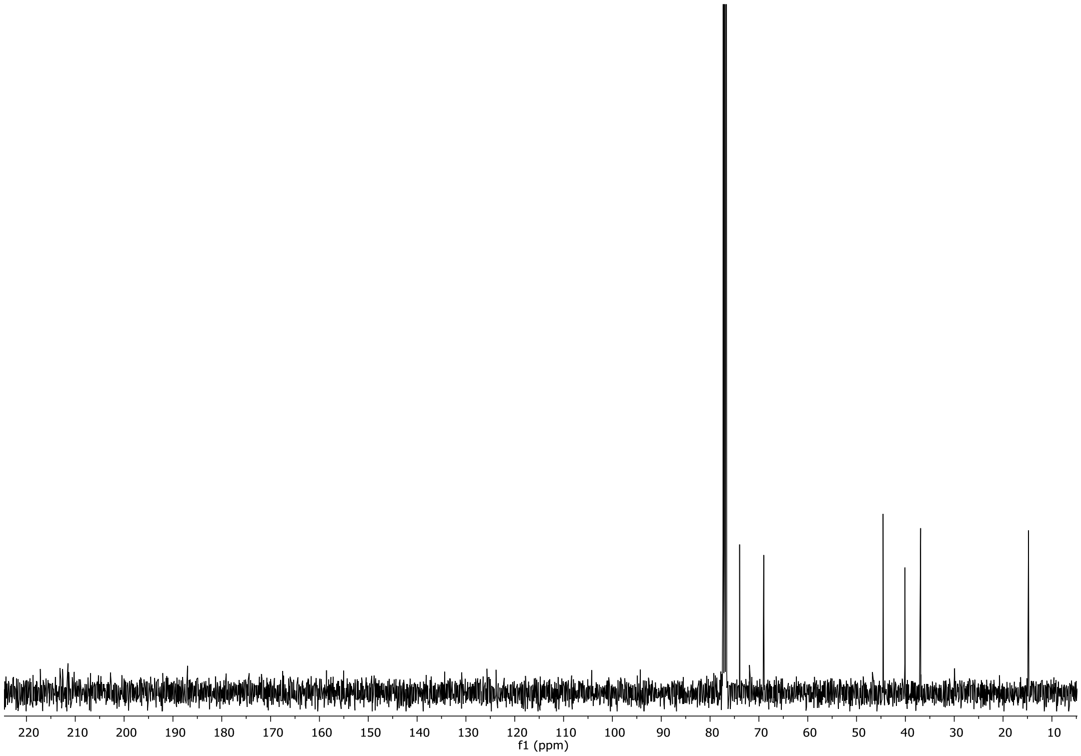

^{13}C-NMR Spectrum of **5** (125MHz, $CDCl_3$)

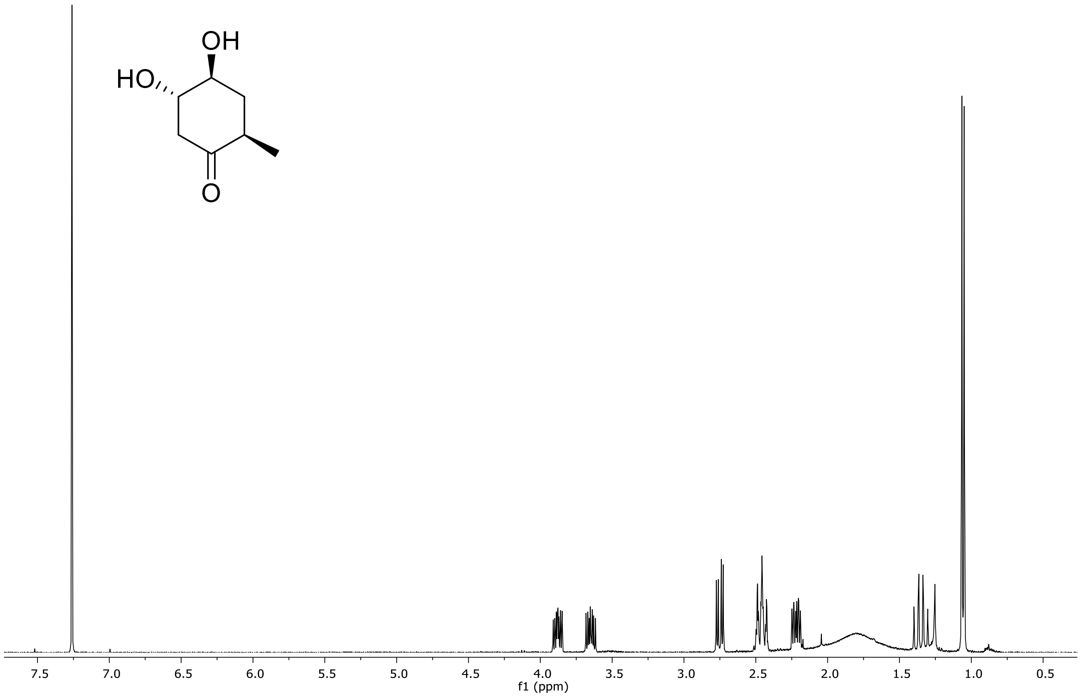

^{1}H-NMR Spectrum of **6** (400MHz, $CDCl_3$)

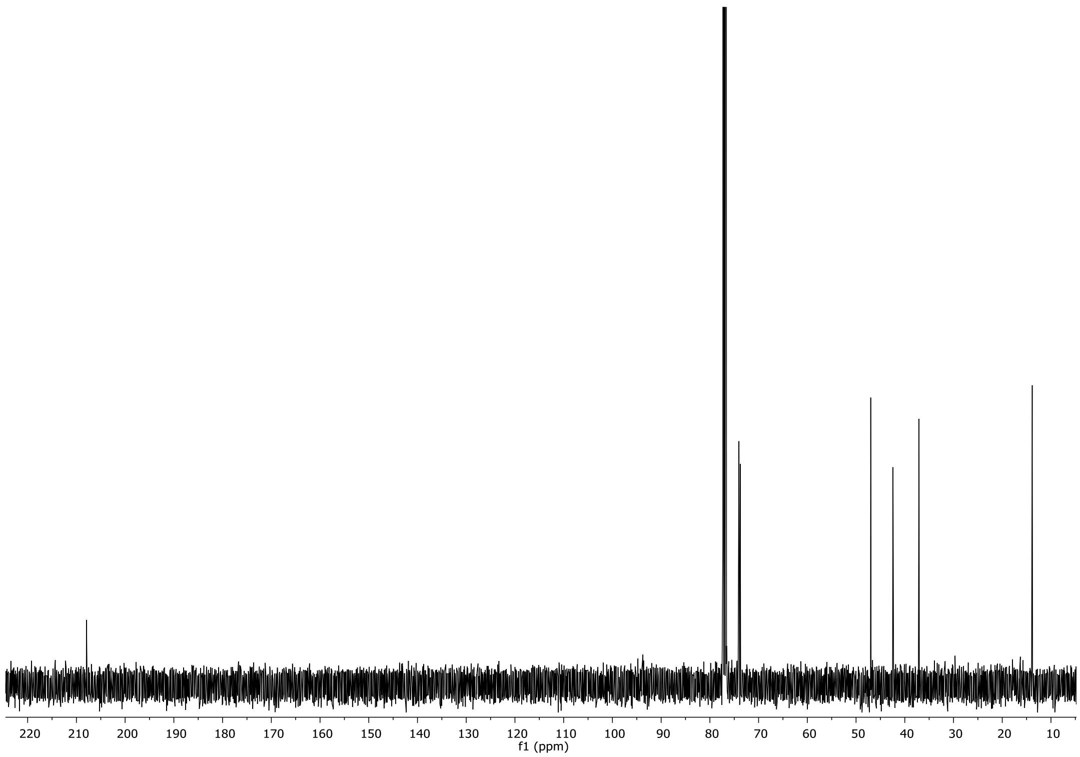

^{13}C-NMR Spectrum of **6** (100MHz, $CDCl_3$)

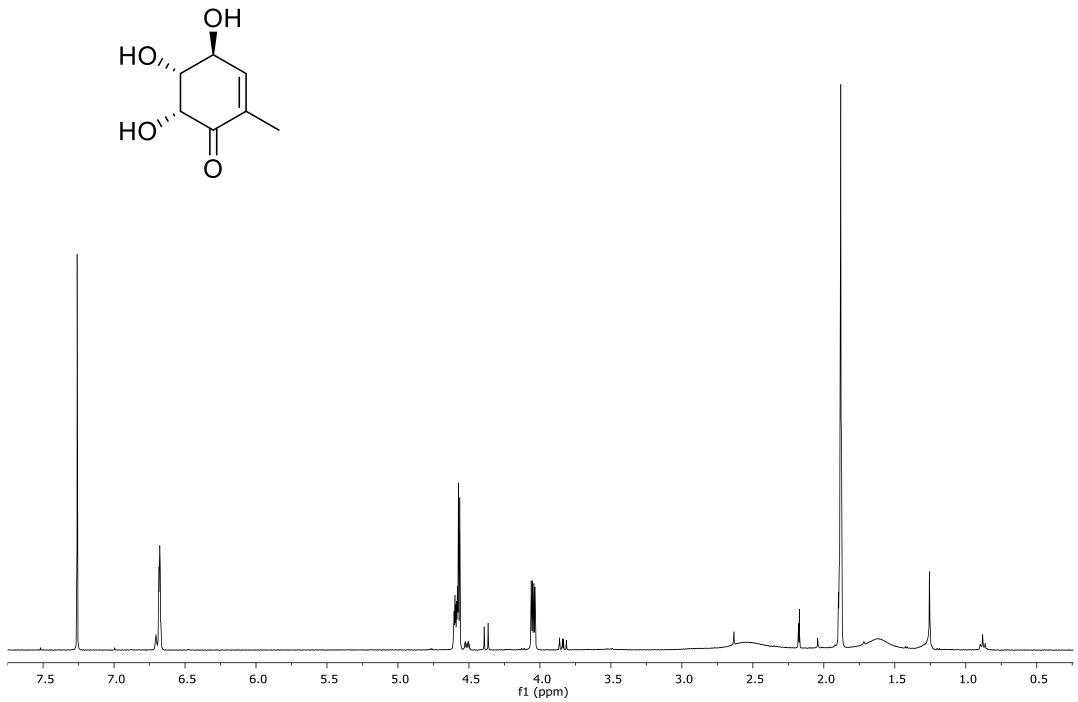

^{1}H-NMR Spectrum of **7** (400MHz, $CDCl_3$)

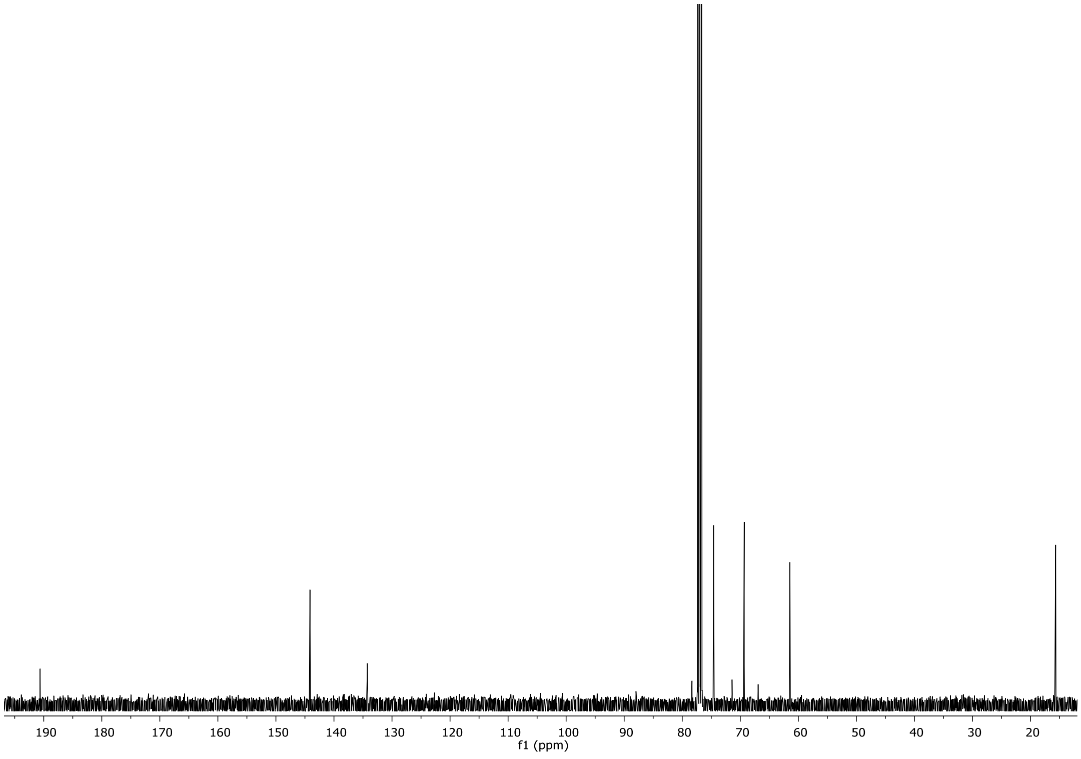

^{13}C-NMR Spectrum of **7** (100MHz, $CDCl_3$)

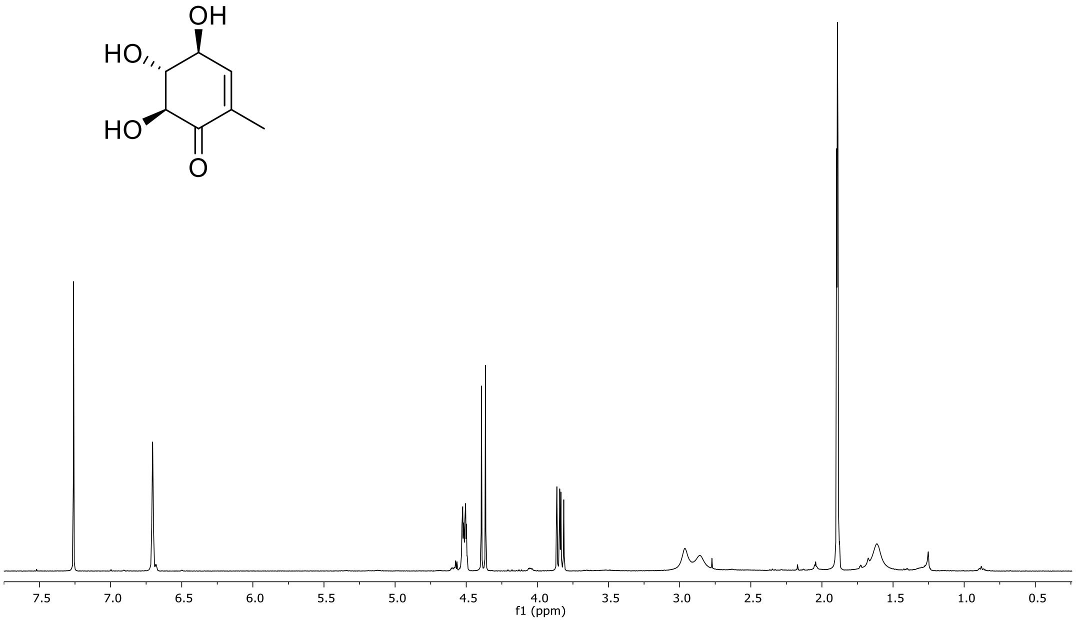

^{1}H-NMR Spectrum of **8** (400MHz, $CDCl_3$)

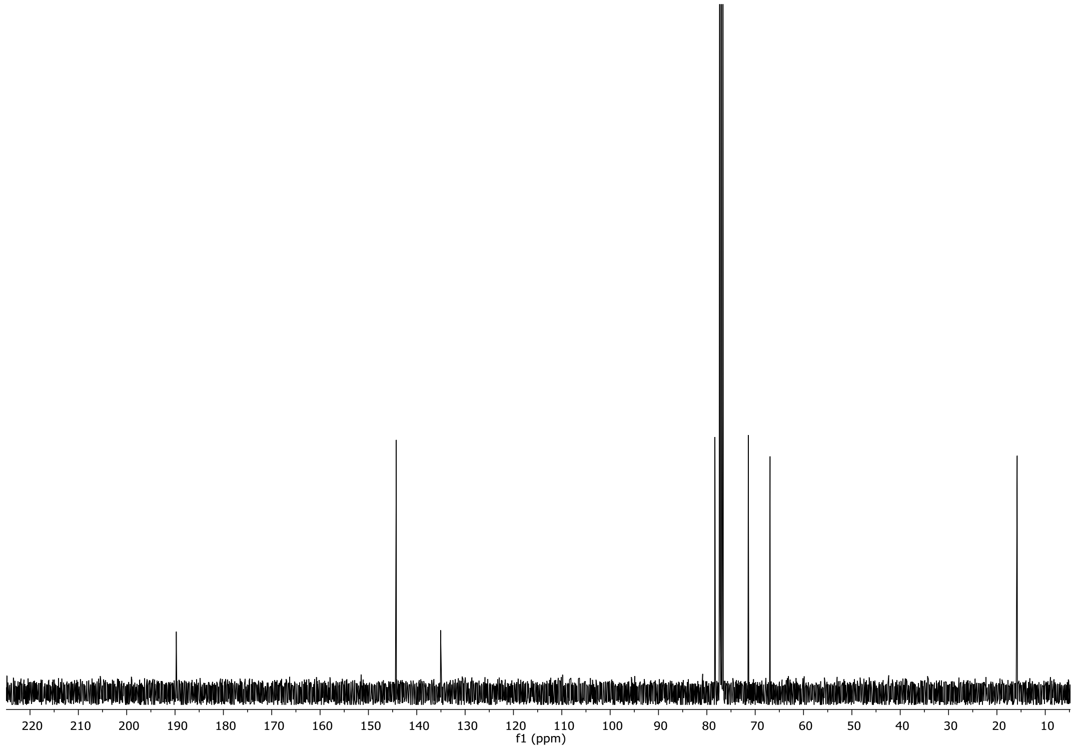

^{13}C-NMR Spectrum of **8** (100MHz, $CDCl_3$)

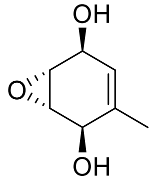

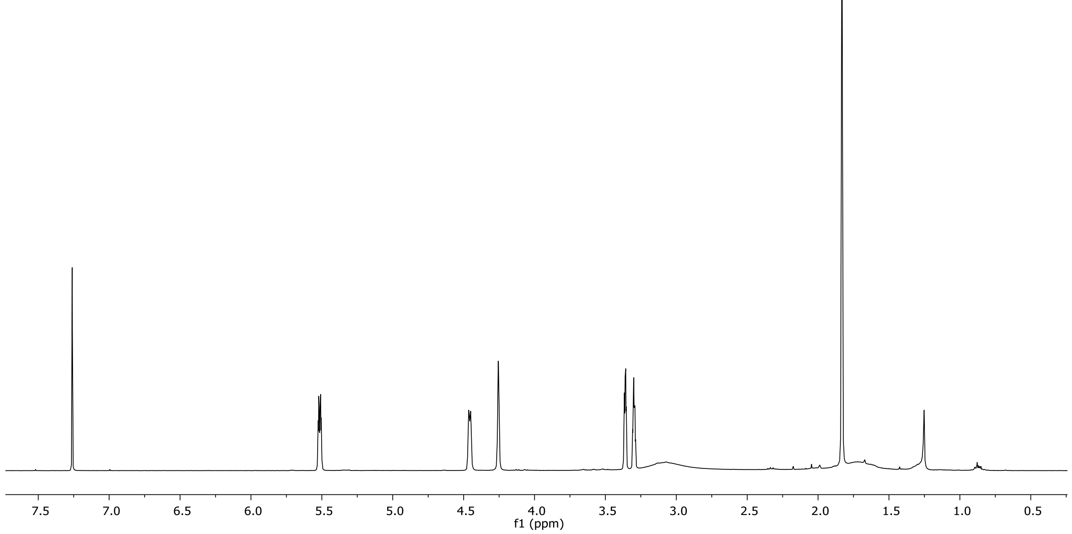

^{1}H-NMR Spectrum of **9** (400MHz, $CDCl_3$)

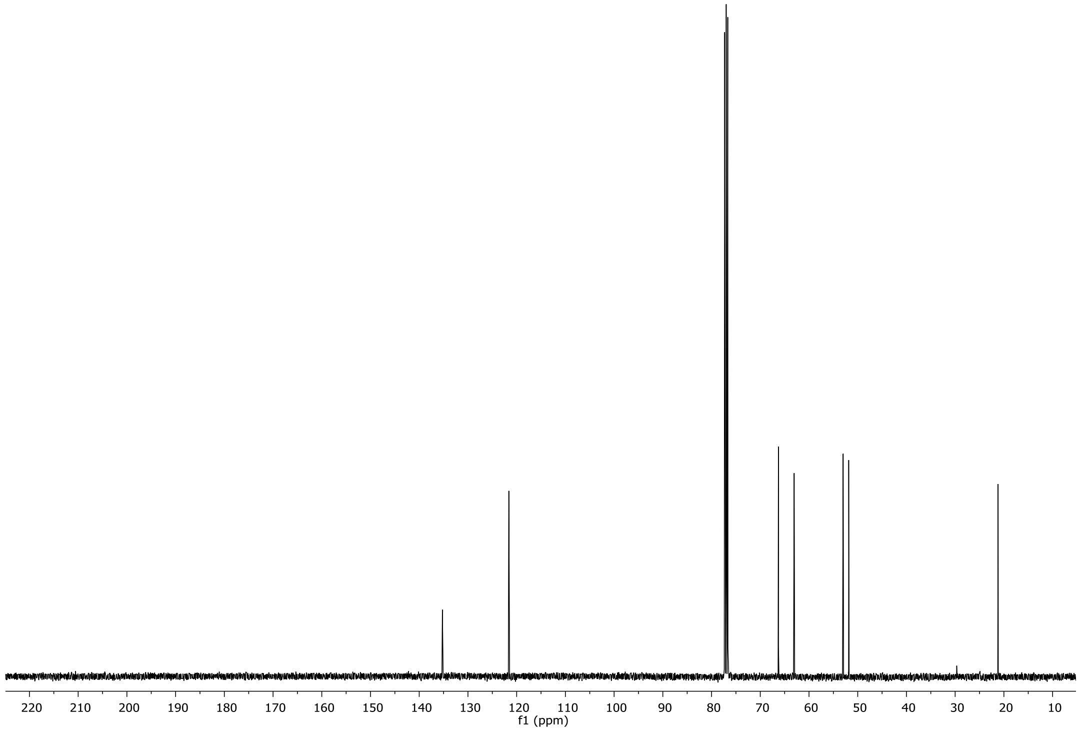

^{13}C-NMR Spectrum of **9** (100MHz, $CDCl_3$)
